# Outcomes of early versus delayed initiation of extracorporeal life support in cardiac surgery

**DOI:** 10.1186/s13019-019-0950-7

**Published:** 2019-07-04

**Authors:** Min Ge, Tuo Pan, Jun-Xia Wang, Zu-Jun Chen, Dong-Jin Wang

**Affiliations:** 1 Department of Cardio-Thoracic Surgery, Nanjing Drum Tower Hospital, the Affiliated Hospital of Nanjing University Medical School, Number 321 Zhongshan Road, Nanjing, 210008 Jiangsu China; 2 Department of Intensive Care Unit, Peking Union Medical College and Chinese Academy of Medical Sciences, Fuwai Hospital, Number 167 Beilishi Road, Beijing, 100037 China

**Keywords:** Extracorporeal membrane oxygenation, Cardiogenic shock, Cardiovascular surgery, Postoperative intensive care

## Abstract

**Background:**

Extracorporeal membrane oxygenation offers temporary hemodynamic support for patients with refractory cardiogenic shock after cardiovascular surgery. However, the initiation time for such patients is controversial. Changing the initiation time might improve the outcomes. This study aimed to investigate whether early extracorporeal membrane oxygenation could improve postoperative outcomes in patients at a high risk of cardiogenic shock.

**Methods:**

In this retrospective study, 173 patients with cardiovascular diseases at a high risk of refractory cardiogenic shock which assessed via empirical risk evaluation from 2010 to 2017 were included. After propensity matching, 36 patients, who were matched to patients initiated with extracorporeal membrane oxygenation after cardiovascular operation (delayed extracorporeal membrane oxygenation group, *n* = 36), were also initiated with such early in the operating room (early extracorporeal membrane oxygenation group, *n* = 36). The primary outcome was death. The secondary outcomes included receiving continuous renal replacement therapy, ventricular arrhythmia, and pulmonary infection.

**Results:**

The demographic and baseline variables were similar between the matched groups. The early extracorporeal membrane oxygenation group showed lower mortality (69.44% vs 41.67%, *P* = 0.03), pulmonary infection morbidity (86.11% vs 55.56%, *P* < 0.01), and continuous renal replacement therapy rate(88.89% vs 66.67%, *P* = 0.04). Moreover, they showed improved cardiac index (*P* = 0.01) and lactate clearance (*P* < 0.01).

**Conclusions:**

Extracorporeal membrane oxygenation provides effective support for cardiogenic failure refractory to medical management; early initiation improves cardiac output and promotes lactate clearance, thus increasing survival in patients with cardiogenic shock after cardiovascular surgery.

**Trial registration:**

This is a retrospective study. It was not registered.

## Background

The incidence of postcardiotomy myocardial dysfunction is approximately 3–5% after cardiac surgical procedures [1]. Extracorporeal membrane oxygenation (ECMO) is the most potent form of acute cardiorespiratory support available and enables complete relief of cardiac workload. ECMO, first introduced clinically in 1972, has been applied as a means of cardiopulmonary support in patients with potential reversible cardiac or respiratory failure when conventional medical strategies have been exhausted [2, 3]. Recently, studies have documented the successful use of ECMO for temporary circulatory support in patients with heart failure [4–6].

However, the Extracorporeal Life Support Organization reports a 31% survival in patients with hypoplastic left heart syndrome (HLHS) undergoing stage I Norwood repair who require mechanical support [7]. Moreover, the survival statistics after ECMO support following cardiac surgery (approximately 50% for infants and 15% for adults) have not been very encouraging and have remained static since its inception in 1973 [8–10]. It has become a major topic in the study of improving the survival rate of patients who need ECMO support after cardiac surgery with postoperative heart failure. There have been reports of higher survival rate comparing patients undergoing cardiac surgery whose ECMO support was initiated in the operating room with those initiated in an emergency situation in the postoperative period [11, 12]. However, the optimal time and indications of ECMO initiation in the operating room are not yet clear.

The most common indications of ECMO in patients after cardiovascular surgery include low cardiac output syndrome, inability to be weaned off from cardiopulmonary bypass (CPB), refractory arrhythmias, pulmonary hypertension, refractory cardiogenic shock, bridge to heart transplant, and extracorporeal cardiopulmonary resuscitation [3, 13, 14]. While these indications and/or criterias are well-established and reliable measurements, there may be a lag between what happens during clinical diagnosis and ECMO initiation. An empirical practice has been developed since 2015 in our hospital, which is used to identify patients at a high risk of refractory cardiogenic shock based on the CPB findings, vasoactive-inotropic score (VIS) [15], cardiac index (CI), and arterial lactate level. The ECMO was early initiated in the operating room in those patients at a high risk of developing postoperative refractory cardiogenic shock. Therefore, we compared the outcomes between patients who were delayed initiating of ECMO (delayed ECMO group) and propensity-matched patients who underwent similar surgical procedures but initiated ECMO early (early ECMO group) to determine if early ECMO, based on the present empirical practice was associated with an improved clinical prognosis.

## Methods

### Patients

From 2010 to 2017, 72 patients older than 18 years who required venoarterial ECMO support following cardiovascular surgery were enrolled in Nanjing Drum Tower Hospital, Nanjing. No funding was received for this study. Patients who received ECMO support prior to surgery were excluded. After the study was approved by the ethical committee of Drum Tower Hospital, we reviewed the hospital medical records, nursing records, laboratory database, and cardiac surgical database.

A retrospective review of our institution’s ECMO database identified 36 propensity matched patients who received ECMO for cardiac support after cardiovascular operation from January 1, 2010, to January 1, 2015. These patients were initiated with ECMO once they had long CPB time (CPB time of > 4 h), persistently poor perfusion (arterial lactate level of > 5 mmol/L and urine output of ≤0.5 mL/kg/h for > 2 h) and impaired ventricular function (CI of < 2.2 L/m^2^/min and mean arterial pressure [MAP] of < 60 mmHg for > 2 h) despite optimized intravascular fluid status and large vasoactive-inotropic treatment (VIS of ≥40 for > 2 h). These patients were considered delayed initiating ECMO if more than three attempts to wean off bypass failed (*n* = 20, implemented ECMO in operative room); or they underwent a 2–4 h observation in theater when they remained unstable after weaning off CPB for 3 times (*n* = 7, implemented ECMO in Cardiac ICU); or because of postoperative emergency but without three attempts to wean off bypass (*n* = 9, implemented ECMO in operative room). Therefore, we used these patients to establish a delayed ECMO group (*n* = 36). In addition, there were 7 patients who were initiated on ECMO in the theater. No patients were initiated on ECMO outside of the theatre.

Owing to the high mortality of the delayed ECMO group (25/36, 69.44%), we implemented an aggressive treatment strategy of initiating ECMO since 2015. Early ECMO was initiated in the operating room when patients met the following criterias at the end of CPB: 1) long CPB time (CPB time of > 4 h); 2) large vasoactive-inotropic agents (VIS of ≥40); 3) CI of < 2.2 L/m^2^/min and MAP of < 60 mmHg; 4) arterial lactate level of > 5 mmol/L; and 5) failure in weaning off from CPB. Thereafter, we prospectively collected the matched patients’ data to establish the early ECMO group. For obtaining better control effects, a propensity score-matched group comprising patients initiated with ECMO in the operating room was compared with the delayed ECMO group. Thus, it was not until December 31, 2017, more than 2 years after the aggressive strategies began, that the 36 other matched patients were finally included.

### ECMO technique and postoperative management

The circuit used was composed of ^3^/_8_-in.-internal diameter polyvinylchloride 2-ethylhexyl phthalate free tubing with heparin biocompatible surface coating. The main components included the Bio-Console 560 (Medtronic, Minneapolis, MN) and Rotafow (Maquet, Hirrlingen, Germany) incorporating a centrifugal pump-based console with an ECMO membrane oxygenator (Medtronic Affinity oxygenator, Medtronic and MaquetQuadrox-iD oxygenator, Maquet). The femoral vein and artery were cannulated through a surgical incision using a 24–33-F outflow cannula and 14–21-F inflow cannula (Medtronic). When the patients had left ventricular dysfunction, a 20-F left ventricular cannulation was performed to reduce the preload (Medtronic). Furthermore, a 15–21-F axillary cannulation was used to avoid limb ischemia, provide antegrade flow and potentially higher cerebral oxygen saturation, and decrease the risk of atheroembolization. A water heat exchange system was used to maintain a constant temperature of the blood in the circuit. Blood flow was monitored using a doppler flow probe attached to the arterial tube of the ECMO. After assembly, the system was primed with a total volume of 400 to 600 mL of normal saline and completely debubbled. Blood flow was maintained up to 60 mL/kg/min to avoid severe hemolysis. We usually controlled blood flow at a range from 30 mL/kg/min to 40 mL/kg/min. Oxygen flow (40–60 mL) was adjusted to maintain an arterial oxygen pressure (PaO_2_) of ≥100 mmHg and obtain oxygenation of the mixed venous oxygen saturation at a level of 60–75%. Heparin was administered at a dose of 100 U/kg body to maintain an activated clotting time of 180–200 s and activated partial thromboplastin time of 50–70 s. The hemoglobin level was maintained at 8–12 mg/dL, and the hematocrit level was maintained between 35 and 45%. The platelet count was maintained at > 50,000–80, 000 μL^− 1^ as much as possible.

The mechanical ventilation parameters were adjusted on the basis of the arterial blood analysis findings. The parameters used for the TBird ventilators (CareFusion Corporation, San Diego, CA) and Dräger Savina ventilators (Drägerwerk AG & Co. KGaA, Lübeck, Germany) were as follows: respiratory rate of 10–18 breaths/min, tidal volume of 6–8 mL/kg, positive end-expiratory pressure of 4–8 cmH_2_O, fraction of inspired oxygenof 0.25–1.0, targeting proper PaO_2_ of 100–150 mmHg, and PaCO_2_ of 35–45 mmHg. In the patients with refractory pulmonary hypertension, nitric oxide at 20–40 ppm and/or sildenafil at 25 mg BID were used. The central venous pressure, MAP, and heart rate were routinely monitored to evaluate cardiac function and circulatory volume. The electrolyte level and colloid osmotic pressure (using human albumin) were maintained within the normal range. Nutritional support was provided via nasointestinal tube feeding 24–48 h after ECMO initiation. Total parenteral nutrition was provided when enteral nutrition feeding was poorly absorbed. Continuous renal replacement therapy (CRRT) was initiated to enable fluid removal based on the input-output balance of 10–50 mL/kg/h in cases of renal insufficiency of renal failure (urine output of < 0.5 mL/kg/h for > 2–4 h). Vasoactive-inotropic agents, such as dopamine (2–10 μg/kg/min), dobutamine (2–10 μg/kg/min), and milrinone (0.3–0.8 μg/kg/min), were commonly used. Epinephrine (0.03–0.15 μg/kg/min) and norepinephrine (0.03–0.4 μg/kg/min) can be additionally used for patients showing hemodynamic deterioration. Vasopressin (0.0001–0.0005 units/kg/min) could be used for patients with low vasoplegic shock. Vancomycin and imipenem-cilastatin sodium were administered for early antimicrobial treatment. Antibiotics were then changed on the basis of the positive antimicrobial susceptibility test findings. The intravascular lines were changed every 10 days when the patients developed a fever (axillary temperature of ≥38 °C).

Weaning off was performed when the patients had satisfactory clinical, biochemical, and echocardiographic parameters. Gradually decreasing in flow by 10% every hour to a flow of < 25 mL/kg/min, ECMO was weaned off when the hemodynamic parameters were maintained. Once the patients showed maintained hemodynamic parameters (MAP of > 65 mmHg, CI of > 2.20 L/m^2^/min, arterial lactate level of < 2.0 mmol/L, VIS of < 15, and left ventricular ejection fraction of ≥40%), decannulation was then performed.

### Statistical analysis

For the statistical analysis, IBM SPSS Statistics for Windows version 22 (IBM Corporation, Armonk, NY) was used. Generally, continuous variables were described as means ± SDs or medians (interquartile ranges [IQRs]), as appropriate, and discrete variables as frequencies (n, %).The Student’s *t*-test was used to analyze normally distributed continuous variables; non-normally distributed continuous variables were compared using the Mann-Whitney U nonparametric method. Two groups were analyzed using repeated measures analysis of variance with various post-tests whenever required. Categorical data were compared using the chi-square or Fisher’s exact test, as indicated. *P* values of ≤0.05 were considered to indicate a significant difference.

We acknowledge the possibility of the existence of bias in our study. In an effort to achieve a sound scientific conclusion, we adjusted for an indication bias using a propensity score. This methodology permitted the comparison of patients who underwent early ECMO against delayed ECMO with a similar risk profile (variables were collected when *P* < 0.1 in the univariate analysis, as presented in Table 1). For each patient, we derived the probability of early ECMO compared with the patients having delayed ECMO in a 1:1 ratio matched by the closest propensity score up to a ± 0. 01 difference.

**Table 1:** Demographic characteristics

Variable	Entire cohort prior matching			Propensity-matched cohort		
	Delayed (*n* = 46)	Early (*n* = 127)	*P* value	Delayed (*n* = 36)	Early (*n* = 36)	*P* value
Age (year)	59.11 ± 15.81	63.81 ± 15.64	0.08	57.31 ± 16.90	61.17 ± 12.22	0.27
Gender (male)	24, 52.17%	71, 55.91%	0.66	19, 52.78%	24, 66.67%	0.23
Weight (kg)	72.22 ± 8.03	73.02 ± 8.21	0.57	71.92 ± 8.27	72.31 ± 8.31	0.84
Body mass index (kg/m^2^)	25.09 ± 3.46	25.21 ± 3.61	0.85	24.89 ± 3.44	24.75 ± 4.13	0.88
NYHA class			0.81			0.94
I and II (n,%)	4, 8.70%	13, 10.24%		3, 8.33%	4, 11.11%	
III and IV (n,%)	42, 91.3%	114, 89.76%		33, 91.67%	32, 88.89%	
EuroSCORE	6.85 ± 1.03	7.64 ± 1.24	< 0.01	6.94 ± 1.94	7.11 ± 1.14	0.53
Previous Medical History						
Acute Myocardial infarction (n,%)	8, 17.39%	24, 18.90%	0.82	5, 13.89%	8, 22.22%	0.36
Arrhythmia (n,%)	5, 19.87%	16, 12.60%	0.76	5, 13.89%	2, 5.56%	0.23
Diabetes Mellitus (n,%)	15, 32.61%	44, 34.65%	0.80	11, 30.56%	10, 2.78%	0.79
Chronic Renal Failure (n,%)	1, 2.17%	4, 3.15%	0.74	0	2, 5.56%	0.15
Hyperlipidemia (n,%)	7, 15.22%	26, 20.47%	0.44	7, 19.44%	4, 11.11%	0.33
Hypertension (n,%)	13, 28.26%	36, 28.35%	0.99	11, 30.56%	10, 2.78%	0.79
Liver Disease (n,%)	1, 2.17%	0	0.11	1, 2.78%	0	0.31
Smoking	16, 34.78%	42, 33.07%	0.83	13, 36.11%	11, 30.56%	0.62
Excessive alcohol (n,%)	15, 32.61%	36, 28.35%	0.59	13, 36.11%	8, 22.22%	0.19
Preoperative LVEF (%)	42(36–52)	43(38–52)	0.48	42(36–51)	45(40–52)	0.17
Preoperative LVDd (mm)	62.10 ± 13.27	58.28 ± 12.89	0.21	61.12 ± 13.19	57.86 ± 12.88	0.29
CPB time (minutes)	262(211–298)	277(237–316)	0.07	262(223–306)	264(220–320)	0.87
ACC time (minutes)	229(194–300)	242(220–286)	0.14	227(191–289)	228(212–254)	0.73
Cardiac re-operation (n,%)	1, 2.17%	6, 4.72%	0.45	0	0	–
Cardioplegic solution			0.50			0.41
HTK (n,%)	10, 21.74%	34, 26.77%		7, 19.44%	10, 27.78%	
CB (n,%)	36, 78.26%	93, 73.23%		29, 80.56%	26, 72.22%	
Intraoperative TEE						
LVEF (%)	43.02 ± 7.72	43.28 ± 8.12	0.85	44.42 ± 7.17	42.33 ± 7.91	0.25
LVDd (mm)	58.59 ± 12.34	61.76 ± 10.70	0.12	57.19 ± 12.18	60.53 ± 12.73	0.26
Surgical Procedures			0.08			0.42
On-Pump CABG (n,%)	7, 15.22%	10, 7.87%		4, 11.11%	4, 11.11%	
Off-Pump CABG (n,%)	1, 2.17%	9, 7.09%		1, 2.78%	7, 19.44%	
Valves (n,%)	16, 34.78%	21, 16.54%		12, 33.34%	10, 27.78%	
CABG+ Valves (n,%)	9, 19.56%	42, 33.07%		8, 22.22%	3, 8.33%	
Aortic operation (n,%)	13, 28.26%	45, 35.43%		11, 30.55%	12, 33.34%	

*NYHA* New York Heart Association, *CABG* Coronary artery bypass grafting, *LVEF* Left ventricular ejection fraction, *CPB* Cardiopulmonary bypass, *ACC* Aortic cross clamp, *TEE* Trans-oesophageal echocardiography, *CB* Cold blood cardioplegic solution, *HTK* Histidine-Triptophan-ketoglutalate solution, Mean ± SD, Median (Interquartile Range), *LVDd* Left ventricular end-diastolic diameter

## Results

There were 173 patients participated in this study. After propensity match, 72 patients with similar risk profile (age, EuroSCORE [16], CPB and surgical procedure) were enrolled in this study (36 in the delayed ECMO group and 36 in the early ECMO group). The baseline and demographic variables were not significantly different between the early ECMO group and delayed ECMO group (Table 1). In matched groups, the early ECMO group had the same age (57.31 ± 16.90 years vs 61.17 ± 12.22 years, *P* = 0.27), EuroSCORE (6.94 ± 1.94 vs 7.11 ± 1.14, *P* = 0.53), and CPB time (median: 262 min, IQR: 223–306 min vs median: 264 min, IQR: 220–320 min, *P* = 0.87) as the delayed ECMO group. In addition, the sex, preoperative left ventricular ejection fraction, preoperative left ventricular end-diastolic diameter, New York Heart Association class, and medical history were also similar between the early ECMO group and delayed ECMO group. The detailed variables are presented in Table 1.

As shown in Table 2, the early ECMO group had a significantly decreased mortality compared with the delayed ECMO group (69.44% vs 41.67%, *P* = 0.03). The early ECMO group also had decreased rates of CRRT use (88.89% vs 66.67%, *P* = 0.04) and pulmonary infection (86.11% vs 55.56%, *P* < 0.01). The rate of successful ECMO weaning was higher (30.56% vs 63.89%, *P* < 0.01), and the ECMO duration for the survivors was shorter (median: 7 days, IQR: 4–10 days vs median: 5 days, IQR: 4–8 days, *P* = 0.03) in the early ECMO group than in the delayed ECMO group. The early ECMO group also had shorter mechanical ventilation time (median: 11 days, IQR: 9–14 days vs median: 9 days, IQR: 7–12 days, *P* = 0.01) and ICU stay for the survivors (median: 31 days, IQR: 21–42 days vs median: 16 days, IQR: 13–54 days, *P* = 0.04). Compared with delayed ECMO group, early ECMO group had lower frequency of pulmonary infections (31, 86.11% vs 20, 55.56%, *P* < 0.01). There were 16 patients who met the same criteria as the early ECMO group but did not receive ECMO from 2010 to 2017. They all died within 7 days after surgery. These 16 patients were implemented Intra-aortic ballon pump (IABP) support (*n* = 9) or IABP+CRRT (*n* = 4). Additionally, no patients had axillary bleeding; no patients were bridged to durable ventricular assist device or transplant in each category; no patient had any paravalvular leak in propensity matched cohort.

**Table 2:** Primary and secondary outcomes in propensity-matched patients

Variable	Delayed ECMO (*n* = 36)	Early ECMO (*n* = 36)	*P* value
Primary outcomes (n, %)			
Death	25, 69.44%	15, 41.67%	0.03
Secondary outcomes (n, %)			
Thromboembolic disease	3, 8.33%	0	0.24
CRRT	32, 88.89%	24, 66.67%	0.04
ARDS	6, 16.67%	4, 11.11%	0.74
Ventricular arrhythmia	5, 13.89%	2, 5.56%	0.43
Femoral bleeding	12, 33.33%	9, 25%	0.60
Gastrointestinal bleeding	7, 19.44%	4, 11.11%	0.51
Limb ischaemia	7, 19.44%	3, 8.33%	0.31
Pulmonary infection	31, 86.11%	20, 55.56%	< 0.01
Other outcomes			
ECMO weaned (n, %)	11, 30.56%	23, 63.89%	< 0.01
MV time (survivor, d)	11(9–14)	9(7–12)	0.01
ECMO duration (survivor, d)	7(4–10)	5(4–8)	0.03
Length of ICU stay (survivor, d)	31(21–42)	16(13–54)	0.04

*ECMO* Extracorporeal membrane oxygenation, *ICU* Intensive care unit, *CRRT* Continuous renal replacement therapy, *ARDS* Acute respiratory distress syndrome, *MV* Mechanical ventilation, Mean ± SD, Median (Interquartile Range)

**Fig. 1:**
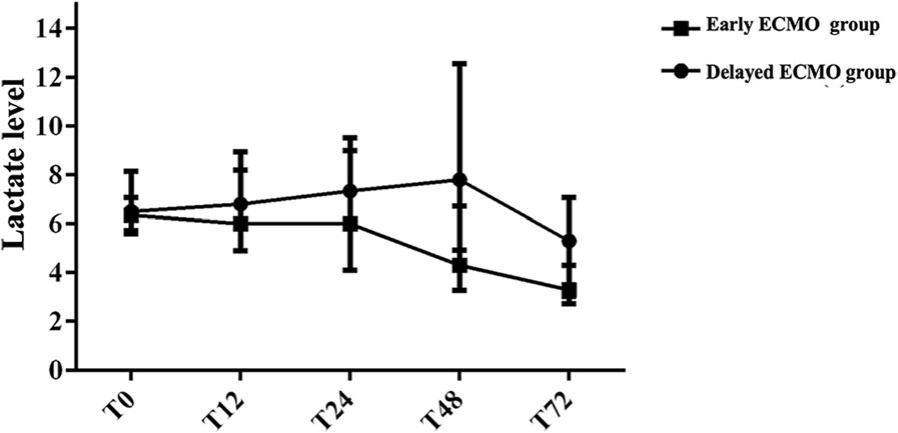
The tendency of arterial lactate during the period of T0 to T72. The early ECMO group had higher rate of arterial lactate clearance compared with delayed ECMO group. Median (interquartile range), *P* < 0.01

From admission to the ICU (T0) to 72 h after surgery (T72), the early ECMO group had a higher rate of arterial lactate clearance (Fig. 1, *P* < 0.01), higher CI (*P* = 0.01), and lower VIS (*P* = 0.03) than the delayed ECMO group. In addition, the ECMO flow and hemoglobin and indirect bilirubin levels had no significant differences between the two groups from T0 to T72. The detailed variables associated with the postoperative hemodynamic variables in the matched patients are presented in Table 3.

**Table 3:** Hemodynamic and related data in propensity-matched patients

Variable	Delayed ECMO (*n* = 36)	Early ECMO (*n* = 36)	*P* value
Arterial Lactate (mmol/L)			< 0.01
Admission to ICU (T0)	6.5(5.6–8.1)	6.3(5.7–7.1)	0.62
T12	6.8(6.0–9.0)	6.0(4.9–8.2)	0.03
T24	7.3(6.1–9.5)	6.0(4.1–9.0)	0.02
T48	7.8(4.9–12.6)	4.3(3.6–6.7)	< 0.01
T72	5.3(3.0–7.1)	3.3(2.7–4.3)	0.03
VIS (maximum)			0.03
T0	52(48–58)	55(48–60)	0.42
From T0 to T12	55(49–58)	55(49–60)	0.35
From T12 to T24	57(50–60)	53(49–60)	0.13
From T24 to T48	56(50–60)	45(40–53)	< 0.01
From T48 to T72	45(35–55)	35(28–45)	< 0.01
CI (L/min·m^2^)			0.01
T0	2.0 ± 0.1	1.9 ± 0.3	0.40
T12	2.0 ± 0.1	1.9 ± 0.18	0.16
T24	1.9 ± 0.2	2.1 ± 0.4	< 0.01
T48	1.9 ± 0.4	2.2 ± 0.3	0.04
T72	2.1 ± 0.4	2.4 ± 0.6	0.06
ECMO flow (ml/kg/min)			0.06
T0	0	40.2 ± 8.4	–
T12	37.3 ± 8.1	32.7 ± 6.1	0.02
T24	36.1 ± 7.6	32.3 ± 6.7	0.03
T48	35.3 ± 7.9	31.1 ± 4.2	0.02
T72	33.5 ± 9.7	31.7 ± 8.7	0.49
Hemoglobin (g/L)			0.27
T0	8.9 ± 1.2	9.4 ± 1.3	0.21
T12	9.50 ± 1.5	9.6 ± 1.1	0.73
T24	9.7 ± 1.4	9.5 ± 1.3	0.42
T48	9.6 ± 1.2	9.8 ± 1.4	0.59
T72	9.5 ± 1.3	9.9 ± 1.2	0.23
Indirect Bilirubin (μmol/L)			0.07
T0	11.8(6.6–18.4)	10.2(6.3–17.2)	0.48
T24	14.8(10.9–21.9)	12.6(9.9–18.5)	0.28
T48	13.0(8.5–20.6)	12.5(5.3–17.9)	0.30
T72	13.1(8.2–19.4)	8.4(5.0–16.2)	0.06
Time since ACC came off to the start of ECMO (minutes)	74(39–143)	29(23–32)	< 0.01

*VIS* Vasoactive-inotropic score, *CI* Cardiac index, *ECMO* Extracorporeal membrane oxygenation, *ACC* Aortic cross clamp, Mean ± SD, Median (Interquartile Range)

The main difference in the timing of initiation of ECMO between the early ECMO group and delayed ECMO group is the failed CPB wean-off for three times (27 patients in the delayed ECMO group, 75%) and once-failed CPB wean-off (31 patients in the early ECMO group, 86.11%). To determine whether the “failed CPB wean-off for three times or once-failed CPB wean-off,” as the main effects between the early ECMO group and delayed ECMO group, could reduce poor outcomes, we investigated the outcomes between the “failed CPB wean-off for three times” in the delayed ECMO group (α group, *n* = 27) and “once-failed CPB wean-off” in the early ECMO group (β group, *n* = 31). The results showed that the β group had significantly decreased rates of mortality (92.59% vs 48.39%, *P* < 0.01), CRRT use (92.59% vs 64.52%, *P* = 0.01), and pulmonary infection (85.18% vs 58.06%, *P* = 0.02) compared with the α group. The detailed variables between the α group and β group are shown in Table 4.

**Table 4:** Primary and secondary outcomes in patients who failed in weaning off bypass

Variable	α group (*n* = 27)	β group (*n* = 31)	*P* value
Primary outcomes (n, %)			
Death	25, 92.59%	15, 48.39%	< 0.01
Secondary outcomes (n, %)			
Thromboembolic disease	3, 11.11%	0	0.06
CRRT	25, 92.59%	20, 64.52%	0.01
ARDS	5, 18.52%	1, 3.23%	0.07
Ventricular arrhythmia	5, 18.52%	1, 3.23%	0.07
Femoral bleeding	7, 25.93%	9, 29.03%	0.79
Gastrointestinal bleeding	6, 22.22%	4, 12.90%	0.34
Limb ischaemia	6, 22.22%	3, 9.68%	0.19
Pulmonary infection	23, 85.18%	18, 58.06%	0.02

*ECMO* Extracorporeal membrane oxygenation, *ARDS* Acute respiratory distress syndrome, *CRRT* Continuous renal replacement therapy, Mean ± SD, Median (Interquartile Range), α: Three attempts to wean off bypass failed, β: Once failed in weaning off bypass

## Disscussion

It is well known that the aim of ECMO is to unload the heart and decrease its work profoundly. Although ECMO allows a finite time interval for the myocardium to recover from injury, the mortality associated with its use is high. However, a number of recent reports on the initiation of ECMO in the operating room for refractory cardiogenic shock have shown encouraging results [11, 12, 17]. Similarly, our study indicated an association of ECMO initiation in the operating room with less vasoactive-inotropic agent requirement, better heart function recovery, low rate of pulmonary infection, and decreased CRRT use. Thus, the initiation of ECMO, based on our empirical risk evaluation practice, could decrease patient mortality.

ECMO after repair of heart diseases can often reverse profound myocardial dysfunction and correct the severe metabolic consequences of a prolonged low cardiac output. The timing of ECMO support directly impacts survival [17–19]. In the past, patients had to be transferred directly from CPB to ECMO, as they could not be weaned off from the bypass despite multiple attempts over an extended period of observation in the theater. Many reports have identified initiation of ECMO in the operating room as an independent predictor for early mortality. A multi-institutional analysis reported that lengthening the time from surgery to ECMO initiation was not associated with worsening mortality [20]. However, Chaturvedi et al. reported that the higher survival of patients cannulated in the operating room than of patients cannulated in the ICU is because of the early effective support preventing prolonged hypoperfusion and the avoidance of a catastrophic cardiac arrest [17]. The Duke University experience similarly did not find the initiation of ECMO in the operating room to impact survival adversely [21]. Therefore, the optimal timing of ECMO initiation remains controversial.

While it is generally accepted that early institution of VA ECMO support in the context of postcardiotomy cardiogenic shock is preferred before multiorgan failure sets in, VA ECMO is an invasive modality and carries complications, such as bleeding, dissection, stroke, etc. Furthermore, prophylactically use of VA ECMO support can pose immense pressure on health care systems, especial socialist health care systems. Hence every case should be considered on the individual merits and multidisciplinary, including advanced patients’ directives and/or wishes, opinion should be taken into consideration prior to institution of such invasive and costly modalities. That was why an early warning system of ECMO initiation was important and necessary to decrease postoperative mortality and hospitalized cost. We found a significant correlation between early initiation of ECMO and promotion of survival in adult patients with acquired heart diseases after cardiovascular surgery. Thus, the initiation of ECMO, based on our empirical risk evaluation practice, might be useful and eligible for clinicians to advance patients’ wishes if such invasive and costly modalities should be implemented.

Second, the population of their studies contained a large proportion of unplanned ECMO. They lacked data related to the decision-making process prior to initiation of ECMO as well as on the adult patients with acquired cardiovascular diseases. In other words, the conditions of the patients prior to ECMO in this study might be better than those of their patients, which could also influence the postoperative outcomes. What demand add is, sharpening the axe will not interfere with the cutting of firewood, explaining the importance of the preparation. Based on our empirical risk evaluation practice, we methodically implemented ECMO for high-risk patients. This study proved that our risk evaluation practice was useful and effective in improving the survival of patients by initiating early ECMO after cardiovascular surgery. To our best knowledge, no study has attempted to deliver criteria for early ECMO interventions in adult patients after cardiovascular surgery. Therefore, this study may be the first to provide the optimal timing of initiation of ECMO for patients with cardiovascular diseases useful as a basis for cardiac surgeons, anesthetists, and intensive specialists worldwide.

Finally, Swan-Ganz catheters were used in all patients to monitor the CI in the operating room. During ECMO, the net cardiac output is the combination of the patients’ cardiac output and the ECMO output. However, the arterial lactate level, VIS, and ECMO flow decreased more rapidly in the early ECMO group than in the delayed ECMO group from T0 to T72. Meanwhile, the CI increased more rapidly in the early ECMO group than in the delayed ECMO group. Patients who receive early initiation of ECMO, which promotes lactate clearance and decreases the dosage of vasoactive-inotropic agents and ECMO flow, may benefit from an improved cardiac output, thus increasing their survival after cardiovascular surgery. It is possible that a proportion of patients in this study who were “electively” placed on ECMO never needed this form of support and IABP support was enough. However, inotropic and vasoactive drugs were routinely used in intraoperative and postoperative management. Additionally, there were 16 patients who met the same criteria as the early ECMO group but did not receive ECMO from 2010 to 2017. They all died within 7 days after surgery. These 16 patients were implemented IABP support or IABP+CRRT. It may be because of the serious preoperative condition of these patients (mean LVEF 42% ± 8%). The ECMO may be prior to IABP for patients with preoperative left ventricular dysfunction. Compared with preoperative echo-variables, the intraoperative LVEF (43.99 ± 8.21% vs 43.67 ± 7.57%, *P* = 0.16) and LVDd (59.49 ± 13.05 mm vs 59.36 ± 12.49 mm, *P* = 0.43) had not significantly changed in propensity matched patients. Therefore, the cardiogenic shock before going on VA ECMO may be resulted from the poor preoperative condition, such as preoperative left ventricular dysfunction and high risk of the surgery (high EuroSCORE), rather than the selectivity of the procedure. Moreover, Alph and beta group comparison was used to illustrate the high mortality of delayed ECMO. In α group, “failed CPB wean-off for three times” means artificially prolonged timing of ECMO initiation. In β group, “once-failed CPB wean-off” means early timing of ECMO initiation. The biggest difference between α and β groups was whether timing of ECMO initiation had been deliberately prolonged. That is to say, as the variables shown in Table 4, deliberately prolonged ECMO initiation could significantly morbidity and mortality. Thus, we suggested that ECMO should be initiated as early as possible.

Notably, several limitations of our study still exist. This study involved the experience in a single center with a relatively small sample size. This observational study could be influenced by potential biases. We used propensity score matching to avoid these biases. However, we cannot account for the factors that affect assignment to treatment and outcomes but cannot be observed in the matching procedure. Any hidden bias due to latent variables might remain after matching, which could lead to some statistical faults. Furthermore, with this analysis, we remove a large number of patients from the analysis, which may have elevated statistical errors. Some limitations are also inherent in non-randomized studies. Thus, randomized controlled studies are needed to re-confirm our conclusion.

## Conclusions

Extracorporeal membrane oxygenation provides effective support for cardiogenic failure refractory to medical management; early initiation improves cardiac output and promotes lactate clearance, thus increasing survival in patients with cardiogenic shock after cardiovascular surgery.

## Data Availability

Please contact author for data request.

## Abbreviations

CHD: Congenital heart disease
CI: Cardiac index
CRRT: Continuous renal replacement therapy
ECMO: Extracorporeal membrane oxygenation
EuroSCORE: European System for Cardiac Operative Risk Evaluation
VIS: Vasoactive-inotropic score
